# Efficacy of modified FOLFOX6 chemotherapy for patients with unresectable pseudomyxoma peritonei

**DOI:** 10.1007/s10147-019-01592-x

**Published:** 2019-12-10

**Authors:** Sakura Hiraide, Keigo Komine, Yuko Sato, Kota Ouchi, Hiroo Imai, Ken Saijo, Masahiro Takahashi, Shin Takahashi, Hidekazu Shirota, Masanobu Takahashi, Chikashi Ishioka

**Affiliations:** 1 Department of Medical Oncology, Tohoku University Hospital, Aoba-ku, Sendai, Miyagi Japan; 2 Department of Clinical Oncology, Institute of Development, Aging and Cancer, Tohoku University, 4-1, Seiryo-machi, Aoba-ku, Sendai, Miyagi 980-8575 Japan

**Keywords:** Pseudomyxoma peritonei, Systemic chemotherapy, 5-Fluorouracil and oxaliplatin (FOLFOX), Unresectable

## Abstract

**Background:**

Pseudomyxoma peritonei (PMP) is a rare malignancy, and there is insufficient evidence about systemic chemotherapy for this disease.

**Methods:**

We retrospectively evaluated the efficacy and safety of a chemotherapeutic regimen with 5-fluorouracil and oxaliplatin (modified FOLFOX6, mFOLFOX6) for patients with unresectable pseudomyxoma peritonei. Patients who received the therapy between April 2000 and February 2019 at the Department of Medical Oncology, Tohoku University Hospital, were enrolled in this study.

**Results:**

Eight patients were treated with mFOLFOX6. The sites of primary tumor were appendix in six patients, ovary in a patient, and urachus in a patient. Six patients received surgery. Seven patients had histologically high-grade PMP, and one patient had low-grade PMP. The median follow-up duration was 27.2 months. All the patients had non-measurable regions as the targets of tumor response. Non-complete response or non-progressive disease was observed in seven patients, with a disease control rate of 87.5%. The median progression-free survival and overall survival were 13.0 months and 27.9 months, respectively. An obvious reduction in the symptoms was observed in two patients. Five patients experienced decline in the serum tumor markers, CEA or CA19-9. The grade 3/4 toxicity that was observed was grade 4 neutropenia in one patient and grade 3 neutropenia in two patients.

**Conclusions:**

mFOLFOX6 might be an effective and tolerable treatment option for patients with unresectable PMP. To our knowledge, this is the first case series of mFOLFOX6 in patients with unresectable PMP and the first case series of systemic chemotherapy for Asian patients with unresectable PMP.

**Electronic supplementary material:**

The online version of this article (10.1007/s10147-019-01592-x) contains supplementary material, which is available to authorized users.

## Introduction

Pseudomyxoma peritonei (PMP) is a rare clinical manifestation of malignancy, characterized by intraperitoneal dissemination of the tumor and progressive accumulation of mucinous ascites with a typical distribution. PMP is also used as a pathologic diagnostic term that is applied to a diverse group of mucinous tumors that have neoplastic cells with various atypia within a background of abundant mucinous deposits.

The histological features of PMP are heterogeneous; therefore, several classification systems have been proposed [1–3]. In the fourth edition of the WHO Classification of Tumors of the Digestive System, PMP is classified as low or high grade based on the histological criteria previously proposed by Bradley et al. [2, 4].

The site of origin of PMP is mostly the appendix; other sites of origin are the ovaries, colon, urachus, and pancreas [1, 5]. Cytoreductive surgery (CRS) combined with hyperthermic intraperitoneal chemotherapy (HIPEC) is recommended as the first-line therapy for patients with resectable PMP worldwide [6]. However, about one-third of the patients with PMP will develop a recurrence after CRS and HIPEC [7, 8]. Moreover, CRS with HIPEC has not been approved, and there is no recommended standard treatment in Japan. More effective treatment options are required for patients with resectable or unresectable/recurrent PMP.

There is limited evidence regarding systemic chemotherapy for unresectable PMP because this disease is quite rare and considered resistant to chemotherapy because of its borderline malignant potential. Considering the recent advances in chemotherapy, it appears important to assess the efficacy of modern chemotherapeutic regimens in unresectable PMP.

Some phase II studies and case series, each from a single-center, show promising results with fluoropyrimidine-based combination therapy [9–13]. Systemic chemotherapy, such as capecitabine plus mitomycin C, 5-fluorouracil (5-FU) and oxaliplatin (FOLFOX4), capecitabine plus bevacizumab, and fluoropyrimidine alone or combination therapy with or without molecularly targeted agents, is reported to help reduction in the volume of tumor and lower the levels of tumor markers in patients with unresectable PMP.

The present single-center, retrospective study was aimed to evaluate the efficacy and safety of 5-FU and oxaliplatin combination therapy, modified FOLFOX6 (mFOLFOX6), that is recently being used more commonly than FOLFOX4, for patients with unresectable PMP, as in the case of metastatic colorectal cancer.

## Patients and methods

Patients with PMP who were treated in the Department of Medical Oncology of the Tohoku University Hospital between April 2000 and February 2019 were enrolled. Their medical records were retrospectively reviewed. Sixty patients were identified with the use of the keywords “pseudomyxoma peritonei”, or “appendiceal carcinoma”, “malignant appendiceal mucocele”, “urachal cancer”, or “ovarian carcinoma with peritoneal carcinomatosis”. Patients who visited our hospital for a second opinion were excluded. We screened these patients to identify patients with unresectable or recurrent PMP who received systemic chemotherapy.

### Diagnosis and treatment

PMP was confirmed mainly using the histological findings of the surgically resected specimens. If surgical resection was not performed because of the patients’ general condition or other reasons, the diagnosis of PMP was established using the biopsy findings and computed tomography (CT) findings of typical PMP, such as the distribution of mucinous ascites and visceral scalloping [14].

We classified each grade of PMP in all eight patients as per the criteria used by Bradley et al. that low-grade PMP corresponds to adenomucinosis or well-differentiated variant of mucinous adenocarcinoma and high-grade PMP corresponds to mucinous adenocarcinoma except for well-differentiated variants [2].

The disease was judged as unresectable based on the clinical course, operative findings, and general condition. Twelve patients with unresectable PMP who received systemic chemotherapy were identified.

Of the 12 patients, one received tegafur and uracil (UFT) with leucovorin as first-line chemotherapy because he denied intravenous chemotherapy; one received irinotecan-based regimen; one received capecitabine, oxaliplatin, and bevacizumab; and one received mFOLFOX6 as perioperative chemotherapy. The remaining eight patients who were treated with at least three cycles of mFOLFOX6 were further analyzed for clinical outcomes, including efficacy and toxicities.

The patients received an mFOLFOX6 regimen of 85 mg/m^2^ oxaliplatin and 200 mg/m^2^ leucovorin administered as 2-h infusions on day 1 followed by a 400 mg/m^2^-bolus of 5-FU with a 46-h infusion of 2400 mg/m^2^ 5-fluorouracil over days 1 and 2. The chemotherapy regimen was repeated once every 2 weeks until disease progression.

### Evaluation of response and toxicity

The patients’ medical records were reviewed for evidence of clinical and radiographic response; serum tumor markers were assessed to evaluate response. Radiologic tumor assessments were performed at baseline and every 2–3 months during the treatment. Response to chemotherapy was evaluated according to the Response Evaluation Criteria in Solid Tumors, version 1.1 [15]. Serum tumor markers were basically assessed once a month. Kaplan–Meier analysis was conducted to estimate median progression-free survival (PFS) and overall survival (OS) using JMP Pro ver. 14.0 (SAS Institute, Cary, NC, USA). OS was calculated from the date of chemotherapy initiation to death or the last follow-up. PFS was calculated from the date of chemotherapy initiation to progression, death, or the last follow-up. The Wilcoxon matched-pairs signed-rank test was used to compare the post-treatment changes in tumor markers that were elevated at pretreatment using JMP Pro ver. 14.0. Toxicity was graded according to the National Cancer Institute Common Terminology Criteria for Adverse Events, version 4.0 [16].

## Ethical statement

This study was approved by the Ethics Committee of the Tohoku University Hospital.

## Results

### Patient population

Patient characteristics are summarized in Table 1. The median age was 59 y (range, 32–77 years). The sites of primary tumor were as follows: the appendix in six patients, ovary in one patient, and urachus in one patient. Two patients had pleural carcinomatosis as well as peritoneal carcinomatosis. Six of the eight patients underwent surgery and were diagnosed with PMP based on the pathological findings. Two patients were diagnosed with PMP based on the biopsy findings and CT findings of typical PMP. Seven patients had histologically high-grade PMP, and one patient had low-grade PMP. No patient received prior systemic chemotherapy or surgery within 28 days before treatment initiation.

**Table 1:** Clinicopathological characteristics of eight patients with PMP who received mFOLFOX6

Patient	Age, Sex	PS	Site of primary tumor	Pathological grade	Extraperitoneal disease^a^	Prior surgery	Best response	PFS (months)	Treatment after mFOLFOX6	OS (months)
1	36, M	0	Appendix	High	(−)	(+)	Non-CR/Non-PD	10.1	BSC	19.1, DOD
2	76, F	1	Ovary	High	(−)	(+)	Non-CR/Non-PD	18.2	FOLFIRI, wPTX	26.5, DOD
3	77, F	0	Appendix	Low	(−)	(+)	Non-CR/Non-PD	31.7	BSC	33.1+ , AWD
4	32, M	2	Appendix	High	Pleura	(−)	Non-CR/Non-PD	10	FOLFIRI + bevacizumab RT for bone metastasis	23.4, DOD
5	62, M	0	Appendix	High	(−)	(+)	Non-CR/Non-PD	14.8	FOLFIRI, TAS102, regorafenib	60.9, DOD
6	69, F	0	Appendix	High	Pleura	(−)	Non-CR/Non-PD	11.2	FOLFIRI + ramucirumab	25.8 + , AWD
7	41, M	0	Urachus	High	(−)	(+)	PD	1.2	FOLFIRI, wPTX	27.9, DOD
8	56, F	0	Appendix	High	(−)	(+)	Non-CR/Non-PD	86.0	Observation	86.7 +, AWD

*M* male*, F* female*, PS* performance status, *mFOLFOX6* 5-fluorouracil and oxaliplatin*, Non-CR/Non-PD* non-complete response or non- progressive disease, *PFS* progression-free survival, *BSC* best supportive care*, FOLFIRI* 5-fluorouracil plus irinotecan*, wPTX* weekly paclitaxel, *RT* radiotherapy, *TAS102* trifluridine/tipiracil, *OS* overall survival, *AWD* alive with disease, *DOD* dead of disease

^a^Extraperitoneal disease which had been diagnosed at the start of mFOLFOX6

### Tumor response and survival

**Fig. 1:**
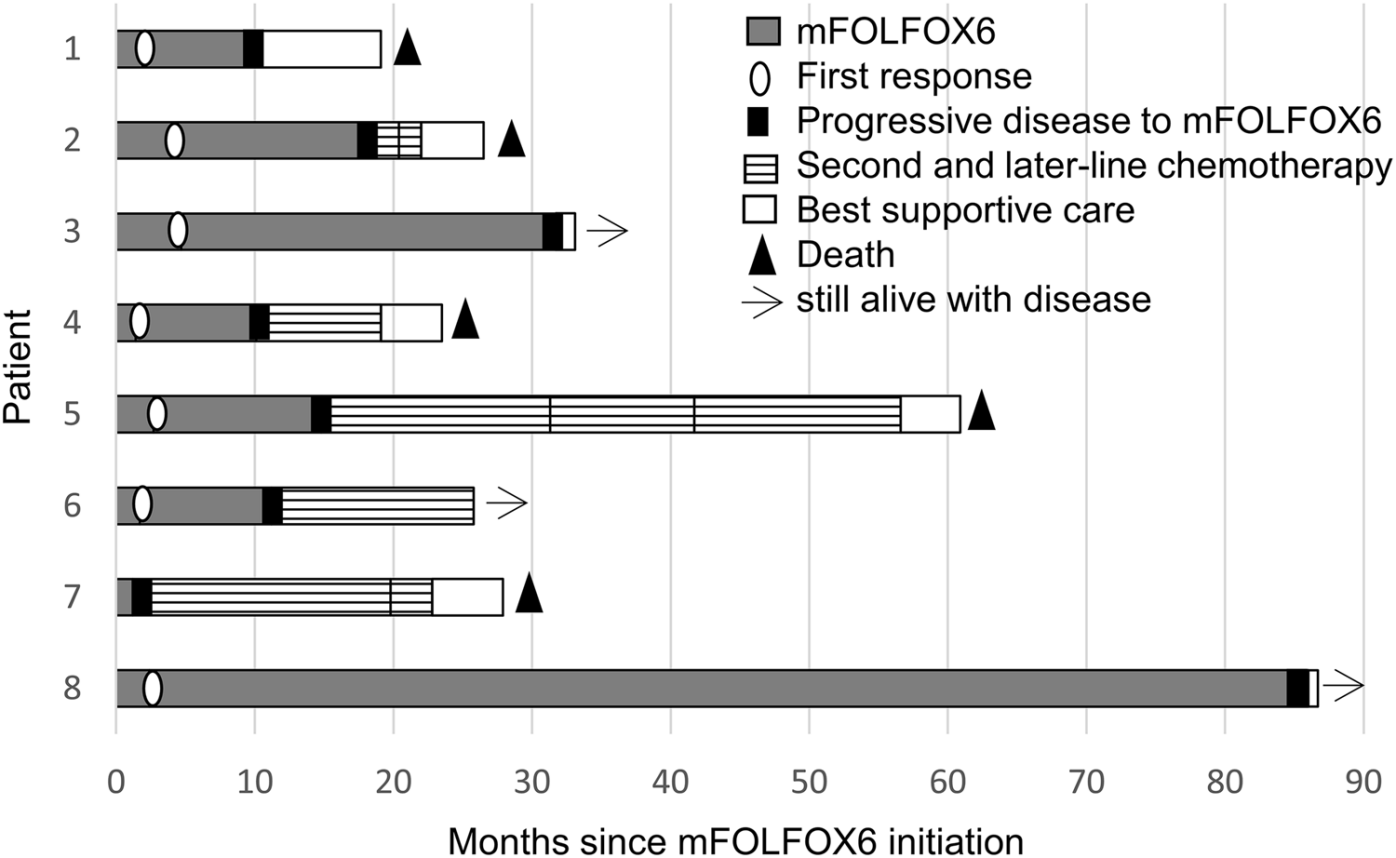
Swimmer plot of the eight study patients. Progression-free survival is represented by a gray segment of a horizontal bar and overall survival is represented by a total segment of a horizontal bar for each patient

All the patients had only non-measurable regions as targets of tumor response. Non-complete response or non-progressive disease (Non-CR/non-PD) was observed in 7 (87.5%) patients, and PD was observed in 1 (12.5%) patient as the best response. The disease control rate (DCR) was 87.5%. After a median follow-up of 27.2 months (range 19.1–86.7 months), all the patients experienced PD. At the time of writing this report, five patients had died, one is receiving second-line chemotherapy, one is under observation, and the other is receiving palliative treatment (Fig. 1). Five patients received second-line chemotherapy, three received up to third-line chemotherapy, and one received up to fourth-line chemotherapy; the regimens included 5-FU plus irinotecan (FOLFIRI), FOLFIRI plus bevacizumab, FOLFIRI plus ramucirumab, paclitaxel, trifluridine/tipiracil, and regorafenib (Table 1).

The median PFS was 13.0 months (95% confidence interval [CI] 1.2–31.7 months), and the median OS was 27.9 months (95% CI 19.1 months to not evaluated owing to censored data). Obvious reduction in the clinical symptoms was observed in two patients after the initiation of chemotherapy. Patient 4 was diagnosed with PMP that originated from the appendiceal mucinous adenocarcinoma based on the CT findings characteristic for PMP and the biopsy finding. The disease extended to the pleura and was thus judged as unresectable. We chose systemic chemotherapy for treatment. We considered that oxaliplatin or irinotecan was inappropriate due to his general condition of Eastern Cooperative Oncology Group Performance Status of 2, with massive pleural effusion and ascites. He received 5-FU plus leucovorin as the first-line treatment after drainage of pleural effusion. After one cycle of 5-FU plus leucovorin, the reduction in pleural effusion was enough to remove a chest drain, and he could be discharged from the hospital. From the 2nd cycle of chemotherapy, oxaliplatin was added, leading to marked decrease in ascites and pleural effusion (Supplementary Fig. 1). He successfully continued to receive mFOLFOX6 for 10 months.

Patient 6 experienced difficulty in consuming oral intake due to intestinal motility disorders caused by compression owing to mass mucinous ascites. After the initiation of mFOLFOX6, her ascites gradually decreased (Supplementary Fig. 2). She was able to take sufficient oral intake and was subsequently free from central venous nutrition after 3 months of mFOLFOX6 therapy.

Patients 3 and 8 experienced slight decrease in ascites even after 12 cycles of mFOLFOX6 (data not shown).

### Tumor marker response

**Fig. 2:**
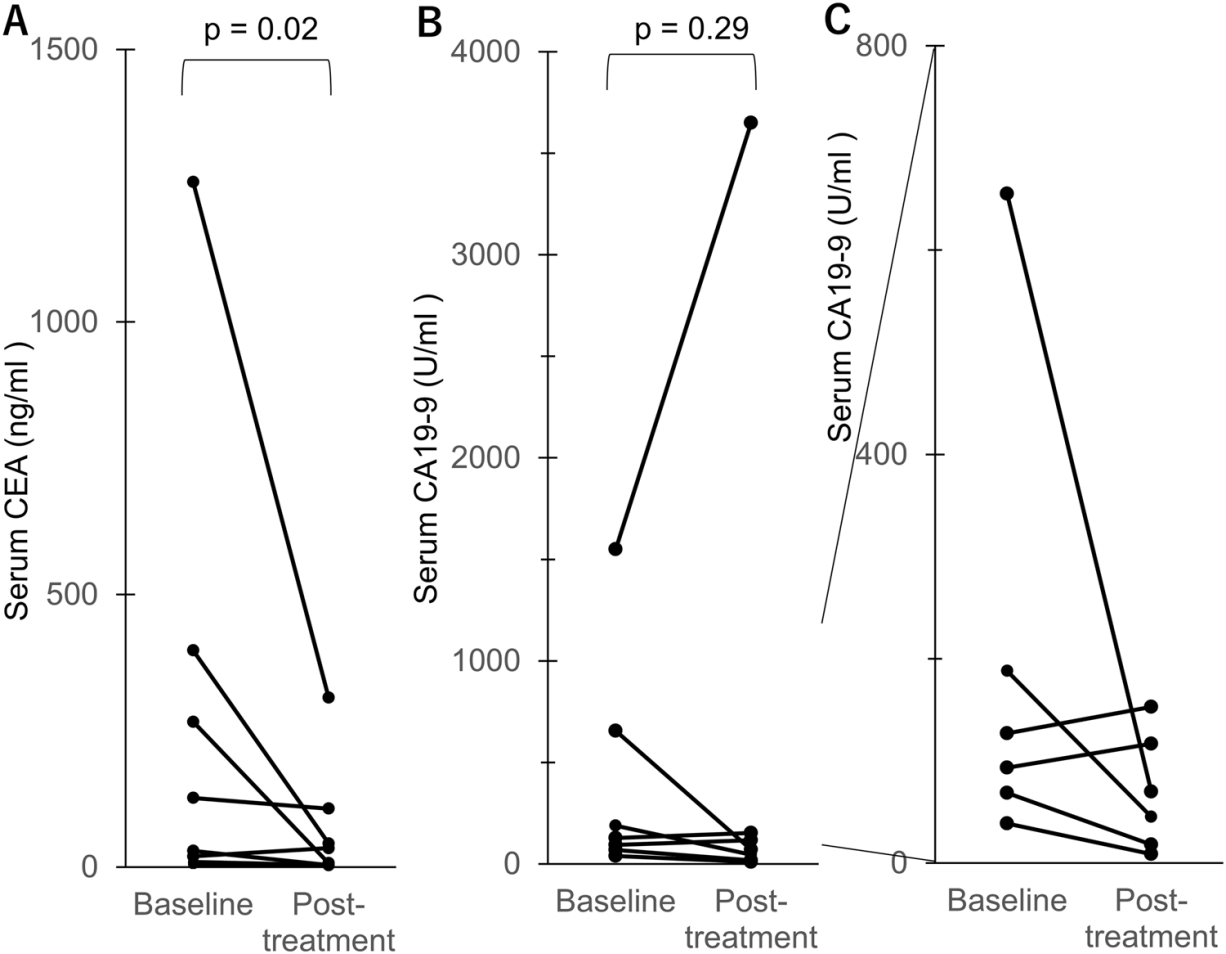
Changes in the serum CEA (**a**) and CA19-9 (**b**) levels from baseline to post-treatment in each patient. The plot shows serum CEA or CA19–9 values for each patient, plotted as a connected line for each patient between pretreatment value and the minimum value during mFOLFOX6 described as “post-treatment”. **c** A magnified view of the values of CA19–9 that were < 800 U/ml in six patients (**c**). Wilcoxon matched-pairs signed-rank test was used to analyze the statistical differences

**Fig. 3:**
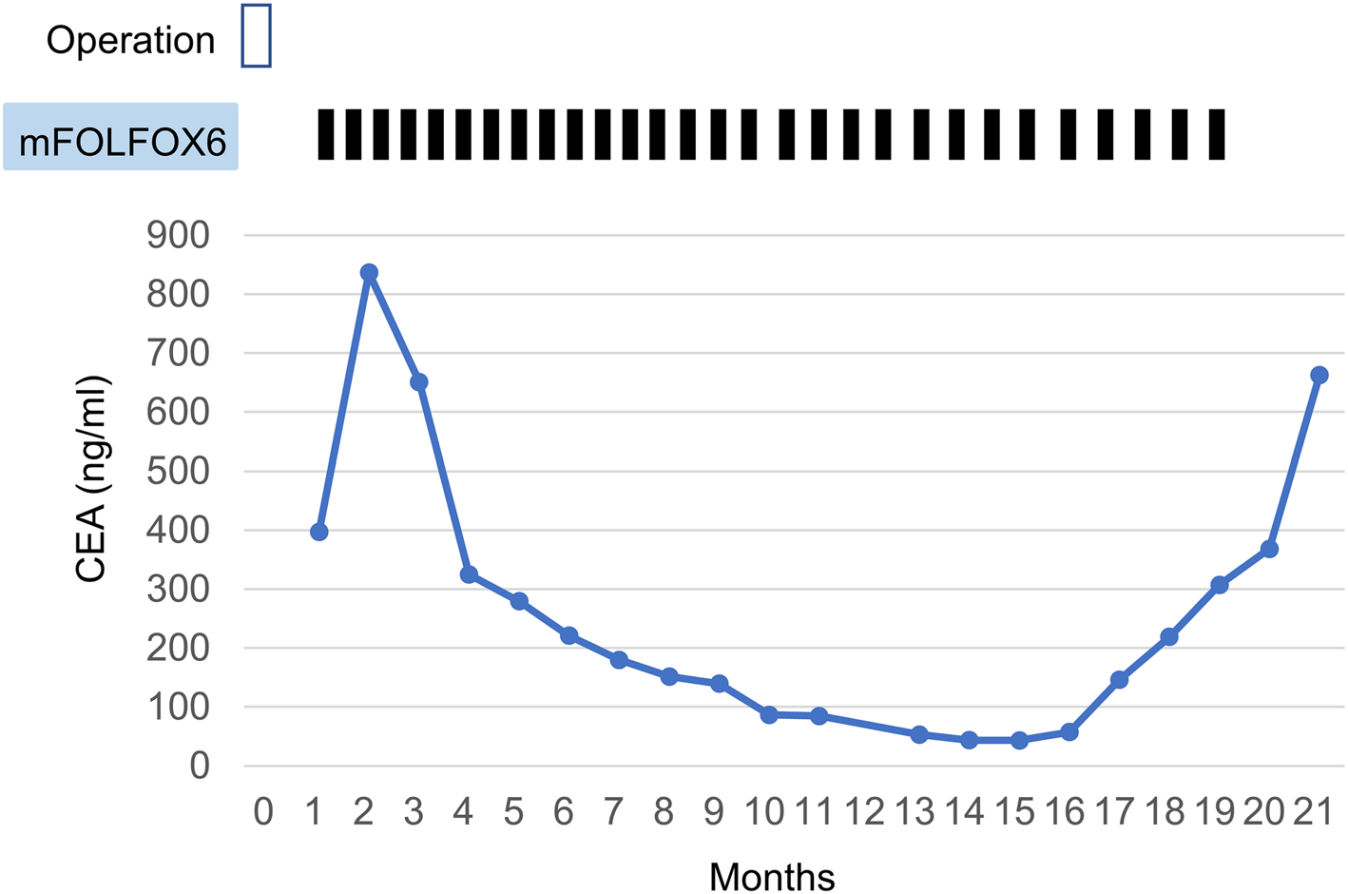
Schematic depiction of the treatment and changes in the serum CEA in patient 2

Serum CEA and CA19-9 was measured for all eight patients. At pretreatment, each serum tumor marker was elevated in all patients, except CA19-9 in patient 4. In five patients, the CEA declined by more than 50% (Fig. 2a), and in four patients, CA19-9 reduced by more than 50% during mFOLFOX6 treatment (Fig. 2b, c). The median CEA level at pretreatment was 78 ng/ml and showed a statistically significant reduction to 21 ng/ml during mFOLFOX6 treatment (*p* = 0.02, using the Wilcoxon matched-pairs signed-rank test). In patient 2, serum CEA continued to decline for a long duration, until up to 23 cycles of chemotherapy (Fig. 3). The median pretreatment CA19-9 of 127 U/mL decreased to 70 U/ml during mFOLFOX6; however, this change was not statistically significant (*p* = 0.29, using Wilcoxon matched-pairs signed-rank test).

### Safety

Overall, 265 chemotherapy cycles were administered, with a median number of 25 cycles per patient (range 3–101 cycles). All hematological and non-hematological toxicities were manageable, and no treatment-related deaths occurred. The grade 3/4 toxicities observed were grade 4 neutropenia in one patient (12.5%) and grade 3 neutropenia in two patients (25%). Febrile neutropenia was not observed. Peripheral sensory neuropathy was observed in all eight patients, including grade 1 in four and grade 2 in four patients. Discontinuation and reintroduction of oxaliplatin described as the OPTIMOX approach [17, 18] was adopted to prevent toxicity progression in patients with grade 2 peripheral sensory neuropathy.

## Discussion

This retrospective analysis demonstrated that some patients with unresectable PMP benefited from mFOLFOX6 treatment. Our data support that mFOLFOX6 is an alternative option for patients who are not optimal surgical candidates. To our knowledge, this is the first case series of mFOLFOX6 for patients with unresectable PMP and the first case series of systemic chemotherapy for Asian patients with unresectable PMP.

Previous studies that have shown PMP to be resistant to systemic chemotherapy were retrospective analyses of treatments performed from 1950 to 1980s [3, 19]. The chemotherapy drugs used in these studies were 5-FU; cyclophosphamide; melphalan; l-phenylalanine mustard; and a combination regimen of semustine, 5-FU, vincristine, and streptozotocin (MOF-Strep). Another retrospective study about treatment conducted from 1996 to 2007 showed that preoperative systemic chemotherapy a poor prognostic factor; however, the authors discussed that a selection bias may have occurred [20]. More recently, since 2000, three studies that analyzed systemic chemotherapy except adjuvant setting in more than five patients with PMP have reported the efficacy of capecitabine plus mitomycin C, FOLFOX4, capecitabine plus bevacizumab, and mainly fluoropyrimidine alone or combination therapy [9–13]. A comparison of these previous studies and the present study is shown in Table 2. Studies for which the number of enrolled PMP patients was unavailable have not been described in Table 2. In a phase II study that evaluated the combination of capecitabine with mitomycin C in 40 patients with unresectable PMP, six patients (15%) demonstrated radiographic reductions and 18 (46%) demonstrated disease stabilization [9]. Pietrantonio et al. reported that the median PFS and OS were 8 months and 26 months, respectively, in a prospective cohort study on 20 patients with unresectable or relapsed PMP treated with FOLFOX4 for a maximum of 12 cycles [10]. In a prospective cohort of 15 patients with relapsed PMP treated with metronomic capecitabine and bevacizumab, the DCR was 87%, and the median PFS was 8.2 months [11]. More recently, a DCR of 87% and a median PFS of 9.5 months was achieved with metronomic capecitabine and cyclophosphamide in a prospective cohort of 23 patients with relapsed PMP [21].

**Table 2:** Previous studies of systemic chemotherapy that included more than five patients with PMP since 2000 and present study

References	Farquharson et al. [9]	Pietrantonio et al. [10]	Pietrantonio et al. [11]	Present study
Study design	Single-center Phase II study	Single-center prospective observational study	Single-center prospective observational study	Single-center retrospective study
Regimen	Mitomycin C + capecitabine	FOLFOX4	Capecitabine + bevacizumab	mFOLFOX6
Number of patients	40	20	15	8
Pathology or pathological grade	DPAM (*n* = 27)^a^ PMCA (*n* = 3)^a^ PMCA-I/D (*n* = 10)^a^	High grade (*n* = 8)^b^ Low grade (*n* = 12)^b^	High grade (n = 5)^b^ Low grade (n = 10)^b^	High grade (n = 7)^b^ Low grade (n = 1)^b^
ORR	Not applicable	20%	20%	not applicable
DCR	Not applicable^c^	65%	87%	88%
Median PFS (months)	Not described	8.0	8.2	13.0
Median OS (months)	2-year OS: 61%	26.2	1-year OS: 91%	27.9

*ORR* overall response rate*, DCR* disease control rate*, PFS* progression-free survival*, OS* overall survival*, FOLFOX* 5-fluorouracil and oxaliplatin, *DPAM* disseminated peritoneal adenomucinosis, *PMCA* peritoneal mucinous carcinomatosis, *PMCA-I/D* PMCA with intermediate or discordant features

^a^Pathological classification described by Ronnett et al. was used [1]

^b^Pathological classification described by Bradley et al. and the WHO Classification of Tumors of the Digestive System was used [2, 4]

^c^The percentage of tumor reduction plus stabilization of progressive disease was 38% [9]

Shapiro et al. retrospectively analyzed the efficacy of systemic chemotherapy with various regimens for patients with unresectable appendiceal cancer with or without PMP; although the exact ratio is unknown, the reported DCR was 55.6% and median PFS was 7.6 months, suggesting the efficacy of chemotherapy even with heterogeneous regimens in appendiceal cancer, irrespective of the presence of PMP [12]. In a single-center retrospective analysis of various systemic chemotherapy with or without molecularly targeted agents for patients with unresectable appendiceal cancer, a median PFS of 9 months and a median OS of 76 months were reported in patients who were administered chemotherapy with bevacizumab; however, the exact ratio of PMP is unknown [13].

It is difficult to directly compare the treatment outcomes of these studies because of the differences in the patients’ characteristics, pre- and/or post-treatments, and methods of tumor response evaluation. Nevertheless, all these reports appear to demonstrate an ORR of about 20%, DCR of about 50–80%, and a median PFS of about 7–8 months, suggesting the efficacy of chemotherapy for PMP to an extent.

Our study achieved efficacy, with a DCR of 87.5% and a median PFS of 13.0 months; thus, our treatment was more efficient than that used in Pietrantonio’s study [10] and other studies [9, 11–13, 21]. The different outcome may be attributable to the cycles of FOLFOX (limitation of a maximum duration of 12 months for treatment in Pietrantonio’s study, and no time limitation and median 25 cycles in our study), the method of drug administration in FOLFOX4 and modified FOLFOX6, and other chemotherapy provided after FOLFOX in some patients. We were unable to evaluate ORR in our study because no patients had measurable lesions. In addition, the pathological distribution of our patients, wherein seven of eight patients had a high histological grade, was different from that in other studies. One of the reasons for this discrepancy might be that patients who were referred to units of medical oncology for a purpose of chemotherapy tend to have cancer with more malignant potential. Patient 3 who had a low histological grade obtained long-term disease control with mFOLFOX6; this result appears to be consistent with previously reported data according to which patients with PMP of low histological grade have better prognosis than those with intermediate or high grade [22].

There is a possibility that post-therapy after FOLFOX affects the OS. Recent studies have reported a favorable outcome of bevacizumab combined with fluoropyrimidine-based chemotherapy [11, 13, 23, 24] and bevacizumab alone or combined with platinum [24, 25]. Choe et al. reported that the addition of bevacizumab to fluoropyrimidine alone or in combination with oxaliplatin or irinotecan improved survival in patients with unresectable appendiceal neoplasms with or without PMP [13]. The median PFS was longer in patients treated with bevacizumab versus that in patients treated without bevacizumab (9 months versus 4 months, hazard ratio 0.69); further, the median OS was also longer in patients treated with bevacizumab (76 months vs. 42 months, hazard ratio 0.49). In our study, two patients received treatment with anti-VEGF agents plus FOLFIRI as second-line chemotherapy. Patient 4 received FOLFIRI plus bevacizumab and patient 6 received FOLFIRI plus ramucirumab as second-line chemotherapy, achieving PFS of 9.0 and 13.9 months, respectively. The OS was 23.4 months in patient 4, and patient 6 was alive at the time of writing this report. There is limited evidence regarding second-line chemotherapy for PMP patients. Despite the difference in the chemotherapeutic regimens and treatment line, patients 4 and 6 obtained comparable PFS with 2nd line therapy in most previous trials [9–13, 21, 23–25]. These reports may suggest that the addition of anti-VEGF agents to chemotherapy, such as FOLFIRI, benefit patients with unresectable PMP. Further studies are needed to assess the benefit of adding molecularly targeted agents, including anti-VEGF agents.

Our study has certain limitations. First, this was a single-institution retrospective analysis. The fact that the natural history of each patient with PMP remains unclear makes it difficult to assess the real effect of treatment because clinical, pathological, and biological features of patients with PMP are heterogeneous. Second, objective assessment of tumor burden was difficult due to multifocal dissemination of mucinous ascites in the peritoneum. Despite these limitations, our results suggest that mFOLFOX6 provides some benefit to patients with unresectable PMP. Symptom reduction in two patients and decline in the serum tumor markers also supported the efficacy of chemotherapy. There may be a possibility of long-term benefit with mFOLFOX6, given that some patients exhibited decrease in ascites or reduction in the serum tumor markers even after 12 cycles of mFOLFOX6 (Fig. 3).

To determine the efficacy of systemic chemotherapy in patients with unresectable PMP, prospective clinical trials are warranted. Given the rarity of this disease, international collaborations of multidisciplinary societies are necessary for conducting clinical trials. In light of the heterogeneity of this disease with respect to the histology ad biology, biomarkers that can lead to optimal approach of treatment in each patient should be developed.

In conclusion, a combination regimen of 5-FU and oxaliplatin including mFOLFOX6 may be an effective treatment option for patients with unresectable PMP. Further studies are warranted to establish more efficient treatment options for patients with this rare malignancy.

## Electronic supplementary material

Below is the link to the electronic supplementary material.

Supplementary file1 (PDF 264 kb)
