# Supplementary material for: Efficacy of modified FOLFOX6 chemotherapy for patients with unresectable pseudomyxoma peritonei
Source: Int J Clin Oncol. 2019 Dec 10;25(4):774–81. doi: 10.1007/s10147-019-01592-x (PMC7118031; doi:10.1007/s10147-019-01592-x)
Supplement: Supplementary file 1 — Supplementary file1 (PDF 264 kb) [file 10147_2019_1592_MOESM1_ESM.pdf]

Efficacy of modified FOLFOX6 chemotherapy for patients with unresectable pseudomyxoma peritonei

International Journal of Clinical Oncology

Sakura Hiraide [1)], Keigo Komine [1)], Yuko Sato [1)], Kota Ouchi [1)], Hiroo Imai [1)], Ken Saijo [1)], Masahiro Takahashi [1),2)], Shin Takahashi [1)], Hidekazu Shirota [1)], Masanobu Takahashi [1),2)], Chikashi Ishioka [1),2)]

[1)] Department of Medical Oncology, Tohoku University Hospital, Aoba-ku, Sendai, Miyagi, Japan

[2)] Department of Clinical Oncology, Institute of Development, Aging and Cancer, Tohoku University, 4-1, Seiryo-machi, Aoba-ku, Sendai, Miyagi 980-8575, Japan

Correspondence author:

Chikashi Ishioka, M.D., Ph.D.

E-mail: chikashi@tohoku.ac.jp

Supplementary Fig. 1

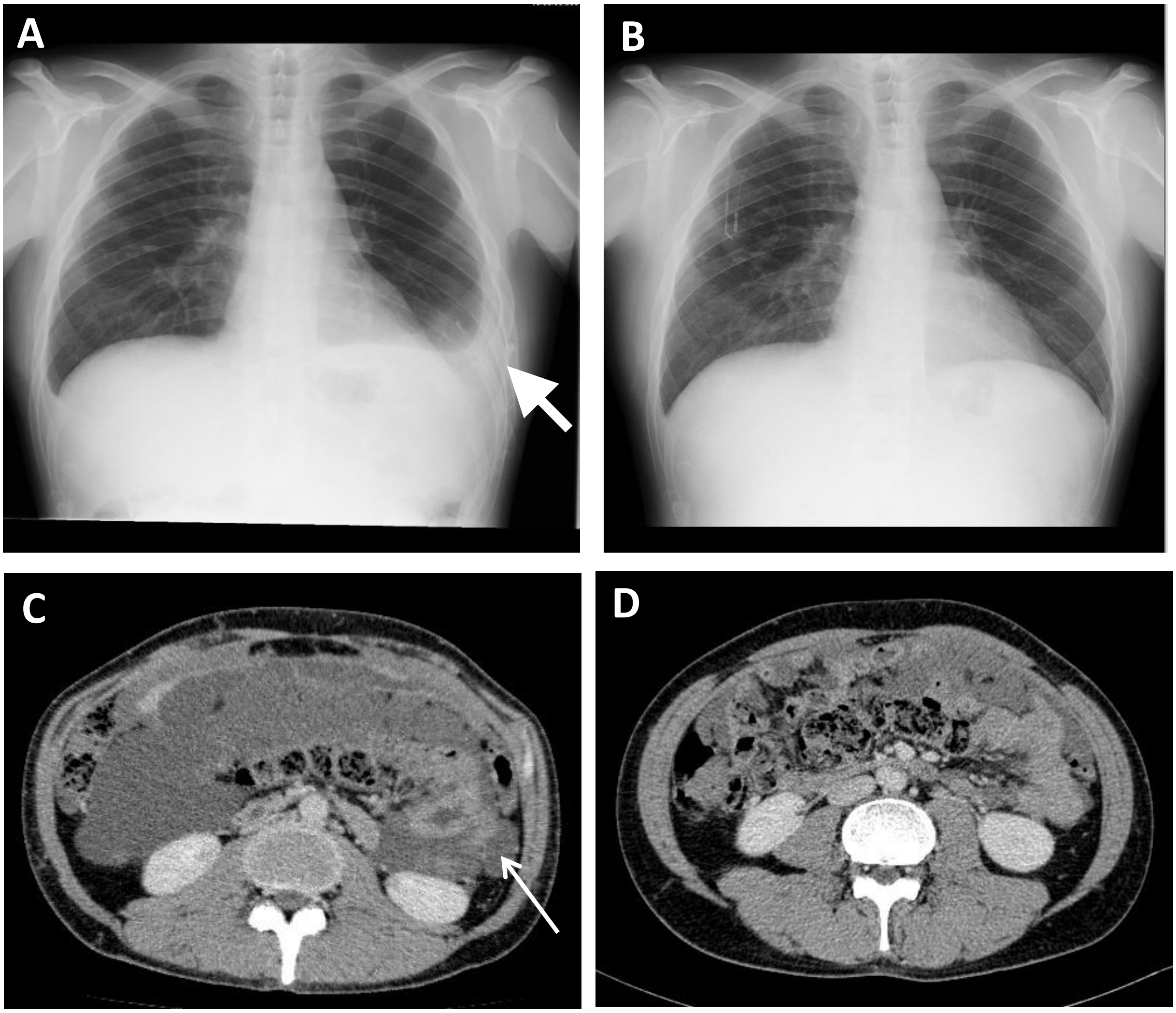

Supplementary Fig. 1 Demonstration of treatment response in patient 4. Pleural effusion (large arrow) shown in the pretreatment chest radiographic image (**a**) decreased 2 months after the initiation of chemotherapy (**b**). Enhanced computed tomography (CT) images 2 months after the treatment (**c**) and images taken 4 months after the treatment reveal reduction in ascites, found obviously in the anterior pararenal space (small arrow) (**d**).

Supplementary Fig. 2

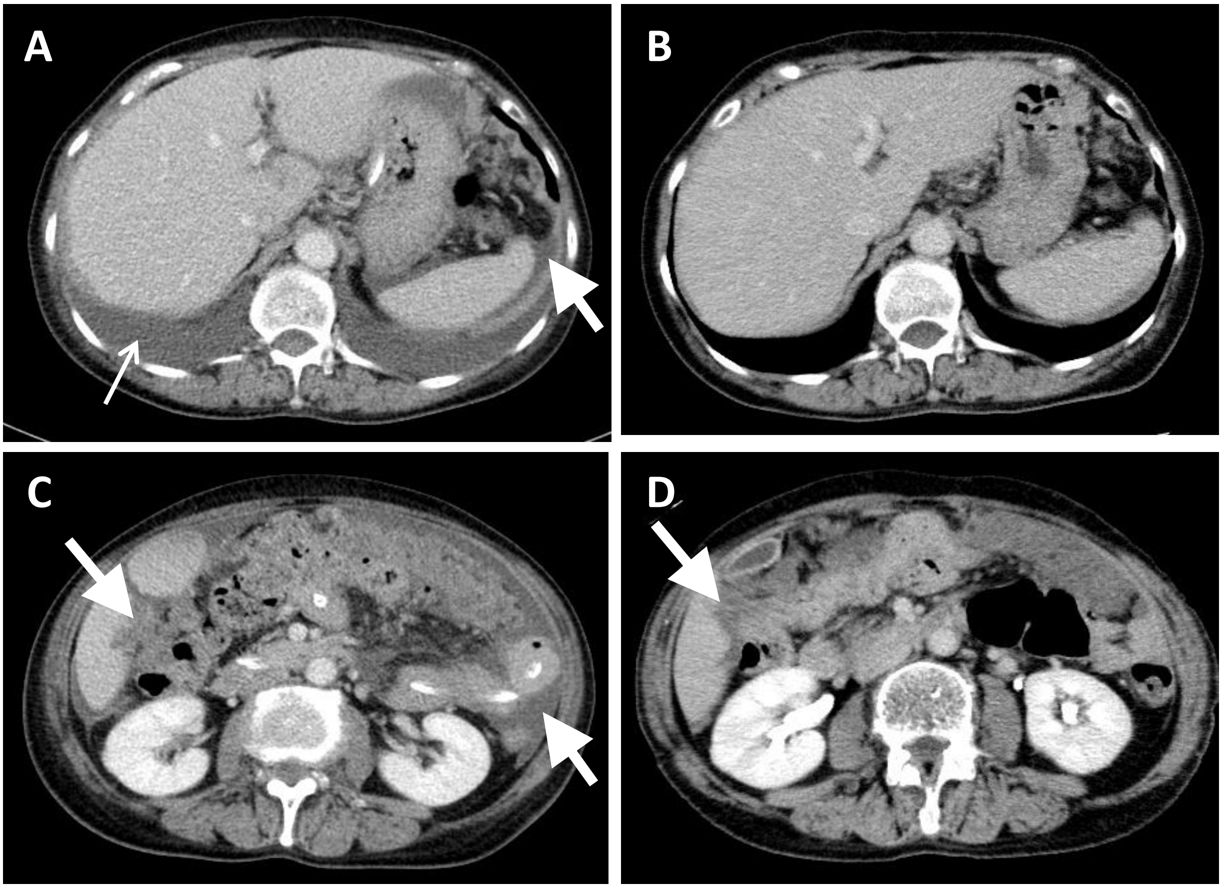

Supplementary Fig. 2 Demonstration of treatment response in patient 6. Pretreatment enhanced CT images show ascites (**a, c**) (large arrow) and pleural effusion (**a**) (small arrow).

Visceral scalloping of the liver, one of the characteristic features of mucinous ascites is indicated by a large arrow. CT images 4 months after mFOLFOX6 show ascites and reduction in the pleural effusion (**b, d**).
